# Molecular Epidemiology of Methicillin-Resistant *Staphylococcus aureus*, Rural Southwestern Alaska^1^

**DOI:** 10.3201/eid1411.080381

**Published:** 2008-11

**Authors:** Michael Z. David, Karen M. Rudolph, Thomas W. Hennessy, Susan Boyle-Vavra, Robert S. Daum

**Affiliations:** University of Chicago, Chicago, Illinois, USA (M.Z. David, S. Boyle-Vavra, R.S. Daum); Centers for Disease Control and Prevention, Anchorage, Alaska, USA (K.M. Rudolph, T.W. Hennessy)

**Keywords:** Staphylococcus aureus, MRSA, Alaska, USA300, community-associated infections, methicillin, epidemiology, genotype, Panton-Valentine leukocidin, research

## Abstract

USA300 is the dominant strain responsible for community-associated (CA) methicillin-resistant *Staphylococcus aureus* (MRSA) infections in most of the United States. We examined isolates from outbreaks of MRSA skin infections in rural southwestern Alaska in 1996 and 2000 (retrospective collection) and from the hospital serving this region in 2004–2006 (prospective collection). Among 36 retrospective collection isolates, 92% carried Panton-Valentine leukocidin (PVL) genes; all carried staphylococcal chromosomal cassette *mec* (SCC*mec*) type IV. None belonged to clonal complex (CC) 8, the CC associated with USA300; 57% were sequence type (ST) 1, and 26% were ST30; 61% were clindamycin resistant. In the prospective collection, 42 isolates were PVL+ and carried SCC*mec* type IV; 83.3% were ST1, 9.5% were ST30, and 7.1% were ST8. Among 120 prospective isolates, 57.5% were clindamycin resistant. CA-MRSA epidemiology in southwestern Alaska differs from that in the lower 48 states; ST8 strains were rarely identified and clindamycin resistance was common.

## Abstract 2

One-sentence summary for table of contents: Epidemiology of MRSA isolates in this region differs from that in the lower 48 states.

*Staphylococcus aureus* is a common cause of skin and soft tissue infections (SSTIs), endovascular infections, pneumonia, septic arthritis, endocarditis, osteomyelitis, foreign-body infections, and sepsis (*1*). In the United States, epidemic infection with community-associated (CA) methicillin-resistant *Staphylococcus aureus* (MRSA) is occurring, with many reports of MRSA infections among persons without traditional healthcare-associated MRSA risk factors (*2**–**4*). As a result, the epidemiology of CA-MRSA has become complex (*5*).

Novel MRSA isolates that are less likely to be resistant to antimicrobial drugs other than β-lactams have been identified in association with epidemic CA-MRSA infections. These CA-MRSA strains are commonly susceptible to drugs such as clindamycin, gentamicin, tetracyclines, and rifampin. Moreover, the genes encoding the pore-forming, bicomponent cytotoxin, Panton-Valentine leukocidin (PVL), are nearly universally present in novel CA-MRSA strains. However, evidence from animal studies has been contradictory in assessing the importance of PVL in the virulence of these isolates (*6**,**7*).

In addition to the PVL genes, strains that cause CA-MRSA infections typically carry staphylococcal chromosomal cassette *mec* (SCC*mec*) types IV and V, small genetic resistance elements that are presumably mobile. A single CA-MRSA genetic background, USA300 (defined by pulsed-field gel electrophoresis), corresponding to sequence type (ST) 8 by multilocus sequence typing (MLST), has become predominant among CA-MRSA isolates in many centers in the United States (*8**–**10*). The reason for the dominance of USA300 is not clear.

Other MRSA strains that are broadly susceptible to non–β-lactams and have PVL genes and SCC*mec* IV or V have predominated among CA-MRSA strains collected in various regions of the world (*11*). In an area of rural Alaska, SSTIs caused by CA-MRSA isolates have been a public health concern since 1996 (*12**–**14*). We explored the molecular diversity of strains causing CA-MRSA in this region and investigated the hypothesis that a transition to the dominance of USA300 had also occurred in this region, similar to that documented elsewhere in the United States (*15*).

## Methods

The study was reviewed and approved by the Institutional Review Boards of the Centers for Disease Control and Prevention (CDC) (Atlanta, GA, USA) and the Alaska Area Native Health Services. The study protocol and draft manuscript were reviewed and approved by the Yukon Kuskokwim Health Corporation (YKHC).

Two collections of MRSA isolates were available for study. They were obtained from the same region of southwestern Alaska. Southwestern Alaska is a roadless area populated principally by Alaska Natives, the descendants of the indigenous people of Alaska. The regional commercial center is Bethel (population 5,700), which is served by the 50-bed Yukon Kuskokwim Delta Regional Hospital (YKDRH) (*13**,**14*). This hospital is the only one serving the 25,000 persons in the YKHC, which is a tribal health corporation that operates a comprehensive system of care in the region for Alaska Natives, including hospital and village-based clinics. Most residents have a subsistence lifestyle, and many homes lack running water. Primary healthcare delivery occurs at village health clinics staffed by community health aides who provide acute and preventive services. Travel between villages is principally by airplane, boat, or snowmobile; no roads connect these regions to the rest of Alaska. The clinical microbiology laboratory at YKDRH serves the hospital and all outpatient clinics in the surrounding region.

### Retrospective Collection

This collection included 36 MRSA strains randomly selected from those obtained during 2 SSTI outbreaks. The 1996 outbreak occurred in 1 village (*12*). In the second outbreak in 2000, MRSA isolates were collected from infections, usually SSTIs, among residents of 29 villages, from wooden surfaces of steam baths in 1 village and from cultures obtained to assess nasal colonization (*14*).

### Prospective Collection

We conducted laboratory-based surveillance for MRSA from January 2004 through January 2006 at the YKDRH in Bethel. During the study period, 85% of all *S*. *aureus* isolates at YKDRH were identified as MRSA (L. Pruitt, pers. comm., January 2008). The first 5 unique-patient MRSA isolates each month were collected by the YKDRH laboratory and sent to the Arctic Investigations Program at CDC for storage and processing. Information accompanying each isolate included basic demographics, location of clinical services, and date and anatomic site of isolation. Isolates were identified at the YKDRH clinical microbiology laboratory. Antimicrobial drug susceptibilities were determined by the Vitek automated system (bioMérieux Vitek, Inc., Durham, NC, USA) at the University of Chicago. Isolates were stratified by patient age and clindamycin susceptibility. Age strata were then compared for differences in the percentage of isolates susceptible to clindamycin by χ^2^ test using Stata SE version 9.2 (StataCorp, College Station, TX, USA).

### Genetic Testing

MLST was performed as described (*16*) at the University of Chicago. Sequence types were grouped into clonal complexes (CCs) when they were genetically related; e.g., ST8 belongs to CC8. CCs were assigned by using the eBURST algorithm as described (*17*). The presence of *mecA* was assessed and the SCC*mec* type of each strain was determined by using criteria previously described (*18*). Presence of *lukF-PV* and *lukS-PV* encoding the PVL toxin was assessed by PCR as described (*19*). Isolates that had these genes were PVL+.

## Results

Thirty-six isolates were available from the retrospective collection. Six were from the 1996 investigation outbreak collected from January 6, 1997, through January 6, 1998, and 30 from the 2000 outbreak collected from April 17, 2000, through September 20, 2000. Of the 2000 outbreak isolates, 3 were collected from steam bath bench surfaces, 12 from nasal survey cultures, and 15 from material obtained from SSTIs. Of 36 isolates in the retrospective collection, 33 (92%) were PVL+ (*14*).

SCC*mec* and MLST typing were performed on the retrospective collection isolates. All carried SCC*mec* type IV. ST1 was the most common MLST genotype, accounting for 20 (57%) of 36 isolates; ST30 accounted for 9 (26%) of 36 and ST59 for 1 (2.8%) of 36. When isolates were grouped in clonal complexes, 22 (63%) belonged to CC1, 11 (32%) to CC30, and 2 (6%) to CC59. None were ST8 or belonged to CC8, the CC most closely associated with USA300 (Table 1).

**Table 1:** Selected characteristics of MRSA isolates from southwestern Alaska, 1997, 2000, and 2004–2006*

Characteristic	Retrospective collection, no. (%)	Prospective collection, no. (%)
Drug susceptibility	n = 36	n = 120
Ciprofloxacin	36 (100)	112 (93.3)
Clindamycin, total†	14 (39)	51 (42.5)
Single-agent testing	33 (92)	51 (42.5)
D-test negative	3/22 (14)	NA
Erythromycin	11 (31)	48 (40.0)
Gentamicin	35 (97)	115 (95.8)
Rifampin	36 (100)	117 (97.5)
Vancomycin	36 (100)	120 (100)
Trimethoprim-sulfamethoxazole	35 (97)	120 (100)
Resistance to >2 non–β-lactam antimicrobial drug classes	21 (58)	69 (58)
PVL genes present	n = 36	n = 42
Yes	33 (92)	42 (100)
No	3 (8)	0
SCC*mec* type IV	36 (100)	42 (100)
MLST CC type		
CC1	22 (63)	35 (83)
ST1	20 (57)	35 (83)
ST1slv‡	1 (3)	0
ST474	1 (3)	0
CC8	0	3 (7)
ST8	0	3 (7)
CC30	11 (32)	4 (10)
ST30	9 (26)	4 (10)
ST484	1 (3)	0
ST535	1 (3)	0
CC59	2 (6)	0
ST59	1 (3)	0
ST59slv‡	1 (3)	0

*MRSA, methicillin-resistant *Staphylococcus aureus*; NA, not applicable; PVL, Panton-Valentine leukocidin; SCC*mec*, staphylococcal chromosomal cassette *mec*; MLST, multilocus sequence typing; CC, clonal complex; ST, sequence type. †Indicates clindamycin susceptibility by single-agent testing and negative D-test results. D-tests for inducible clindamycin resistance were not indicated for any isolates from the prospective collection and were performed for 22 isolates in the retrospective collection. ‡slv, single-locus variant by MLST testing.

Only 14 (39%) of 36 MRSA retrospective collection isolates were susceptible to clindamycin when clindamycin single-agent testing and D-test results were taken into account; 31% were susceptible to erythromycin. Susceptibility to gentamicin, ciprofloxacin, rifampin, vancomycin, and trimethoprim-sulfamethoxazole was nearly universal (Table 1).

Of the 120 MRSA patient isolates available from the prospective collection, 117 were from patients whose sex was known; 61 (50.8%) were from males. Most patients were 13–49 years of age. Among patients with known venue of care, 106 (90.6%) of 117 were outpatients, 59 (50.4%) of whom received care in the emergency department. All but 1 of the 11 inpatients had their isolate obtained within 72 hours of admission, i.e., their infections had onset in the community. Most (90.0%) isolates were from material obtained from SSTIs. Other sites of isolation included blood, urine, respiratory tract, and bone (Table 2).

**Table 2:** Demographic and clinical characteristics for 120 patients infected with MRSA isolates in the prospective collection, southwestern Alaska, 2004–2006*

Characteristic	No. (%)
Sex	
Male	61 (50.8)
Female	56 (46.7)
Unknown	3 (2.5)
Age, y	
>1–2	11 (9.2)
3–12	14 (11.7)
13–20	22 (18.3)
21–49	53 (44.2)
>50	18 (15.0)
Unknown	2 (0.2)
Location of care	
Inpatient	11 (9.2)
Outpatient	106 (88.4)
Emergency department	59 (49.2)
Other outpatient	47 (39.2)
Unknown	3 (2.5)
Place of onset by 72-h rule†	
Community	114 (95.0)
Hospital	1 (0.8)
Unknown	5 (3.3)
Site of isolation	
Blood	3 (2.5)
Respiratory tract	1 (0.8)
Skin/wound	108 (90.0)
Urine	2 (1.7)
Bone/joint	1 (0.8)
Other	5 (4.2)

*MRSA, methicillin-resistant *Staphylococcus aureus*. †MRSA isolates were considered community onset if they were obtained from a patient in the outpatient setting or from a hospitalized patient within 72 h of admission; only 1 isolate was obtained from a patient who was considered to have had onset of the infection in the hospital.

A random sample of one third of the prospective collection isolates (42/120) spanning the entire collection interval were assayed for PVL toxin genes, SCC*mec*, and MLST type. All 42 isolates tested were PVL+, and all carried the SCC*mec* type IV element. Most MRSA isolates in the prospective collection were ST1 (35/42, 83.3%), which likely represent USA400. Other types included ST30 (4/42, 9.5%) and ST8 (3/42, 7.1%). ST8 isolates likely represent USA300 (Table 1).

Antimicrobial drug susceptibility data were available for all 120 isolates in the prospective collection; 51 (42.5%) of 120 were susceptible to erythromycin and clindamycin. There was no discordance in erythromycin and clindamycin susceptibility; therefore, no isolate underwent the D-zone test. Nearly all isolates in the prospective collection were susceptible to ciprofloxacin (93.3%), gentamicin (95.8%), and rifampin (97.5%). All isolates were susceptible to trimethoprim-sulfamethoxazole and vancomycin. More than half (57.5%) of the isolates were resistant to >2 classes of non–β-lactam antimicrobial drugs; most of these were accounted for by isolates resistant to erythromycin and clindamycin (Table 1).

We examined the susceptibility to clindamycin of MRSA isolates from the prospective collection stratified by patient age groups (0–2, 3–12, 13–20, 21–49, >50 years of age). There were no significant differences or trends in the rate of clindamycin resistance among different age strata (p = 0.47).

## Discussion

CA-MRSA isolates from southwestern rural Alaska differed in important ways from isolates collected in other parts of the United States. These isolates almost universally belonged to CC1, with a minor representation in CC30, CC59, and CC8. Elsewhere in the United States, USA300 (belonging to CC8) has become the most common cause of community-associated SSTIs at medical centers in Atlanta, Baltimore, San Francisco, Houston, Chicago, and Los Angeles (*2**–**4**,**8**,**9*) and among adults with SSTIs who came to emergency departments in 17 cities (*8*). In contrast, ST8 (corresponding to USA300) was an infrequent genotype among MRSA isolates from rural Alaska in 2004–2006 and was absent among isolates from 2 outbreaks in separate villages in 1996 and 2000. Despite these differences in genetic background among the CA-MRSA isolates, nearly all isolates tested were PVL+ and all carried SCC*mec* IV.

The molecular epidemiology of CA-MRSA infection in Alaska underscores the worldwide geographic diversity of novel CA-MRSA genetic backgrounds identified in the past decade. Isolates containing the PVL genes and either SCC*mec* IV or SCC*mec* V that lack resistance to most non–β-lactam antimicrobial drugs have been identified in 6 continents. Examples include ST5 in France and Switzerland; ST80 in Belgium, Croatia, Denmark, England, Finland, Germany, Greece, the Netherlands, Norway, Romania, Scotland, Slovenia, and Sweden; and ST22 in Germany and the Netherlands (*11*).

The 3 most common genetic background types in rural Alaska (ST1, ST59, and ST30) have been reported from studies of MRSA in communities elsewhere. ST1, corresponding to pulsed-field gel electrophoresis type USA400, was the strain type responsible for the deaths of 4 children reported in the midwestern United States (*20*) and the type that predominated in Chicago (*10*) and other regions in the late 1990s. The prototype strain is MW2, the genome of which has been sequenced (*21*). ST1 has become a rare cause of SSTIs in Chicago, Texas, and California, and among SSTI patients at emergency departments in 17 US cities (*8**,**10*).

In Taiwan, ST59 is predominant among strains that are PVL+ and carry SCC*mec* IV or V_T_ (*18*). USA1000, which is also an ST59 strain, circulates among persons with no known exposure to the healthcare system (*22*). Some genetic diversity has been noted among ST59 strains as shown by variation in staphylococcal protein A (*spa*) typing (*11**,**23**–**25*). ST59 strains were also isolated at a decreasing frequency in 1997–2001 from patients in a California jail (*15*), and in Western Europe and Singapore (*11*). We found 1 ST59 isolate and 1 single-locus variant of ST59 in the retrospective collection from the 1996 and 2000 outbreaks but found none in the prospective collection.

The evolutionary history of ST30 MRSA strains is complex; the acquisition of the SCC*mec* element and the PVL genes has likely occurred in this genetic background on several occasions. Phage type 80/81 strains of *S*. *aureus*, virulent nosocomial pathogens in the 1950s and 1960s, shared this ST background (*26*). By examining the pattern of resistance-gene carriage in various MRSA genetic backgrounds, Diep et al. proposed an evolutionary relationship among ST30 strains, suggesting that an MSSA ST30 strain sequentially added to its genome phage-encoded PVL toxin genes and the SCC*mec* IV element (*27*). However, a strain of ST30 MRSA isolated in 1991 from Wisconsin lacked the PVL genes but carried SCC*mec* IV (*25*), which suggested that the sequence of events hypothesized by Diep et al. does not universally describe the evolution of these strains (*27*). Among 5 ST30 MRSA isolates collected in Japan in 1979–1985, 3 were PVL+ and all carried the SCC*mec* type I element (*28*). ST30 isolates reported from various regions commonly carry PVL genes and the SCC*mec* IV element but can differ in *spa* type, which suggests a continued and complex evolutionary trajectory for this prevalent sequence type (*8**,**11**,**18**,**24**,**29**–**32*).

The difference in PVL+, SCC*mec* IV strain types of MRSA in rural Alaska compared with those in the lower 48 states suggests that Alaska may represent an earlier part of the epidemic curve of CA-MRSA. For example, there was a shift from USA400 to USA300 as the predominant clindamycin-susceptible, PVL+, SCC*mec* IV-containing MRSA strain in Chicago after 2000 (*10*). The predominance of ST1 strains in southwestern Alaska may reflect geographic isolation of this region or improved fitness of the strain in the rural Alaskan environment. The clinical spectrum of these community-onset cases is similar to MRSA disease elsewhere with a predominance of SSTIs and few associated instances of bacteremia or other invasive illnesses. This disease spectrum is also similar to that of earlier reports of infections caused by MRSA from this region (*13**,**14*). O’Hara et al., using phylogenetic analyses of the *lukSF-PV* sequences coding PVL toxin in a sample of international clinical MRSA isolates, recently hypothesized that USA300 emerged after a CC8 MRSA strain acquired the PVL genes from the preexisting, virulent MW2 strain (*33*). If this event was the genesis of USA300, this event may have occurred in the lower 48 states, and USA300 had not spread to southwestern Alaska, where USA400 strains still predominated in early 2006.

All MRSA isolates we tested carried SCC*mec* type IV. So-called healthcare-associated MRSA isolates typically carry SCC*mec* types II or III, lack PVL genes, tend to be resistant to a greater number of non–β-lactam antimicrobial drugs, and were predominant among strains isolated from cases of hospital infections in the United States before 2000 (*5*). Such healthcare-associated MRSA isolates were absent from our isolate collections, even from the prospective collection, which was a random sample of MRSA isolates that included inpatients in the region served by the hospital laboratory for 2 years. In contrast, at the University of Chicago in 2004–2005, 8.6% of MRSA isolates from pediatric infections and 51.7% from adult infections carried SCC*mec* II (*5*).

The PVL+, SCC*mec* IV–bearing strains of MRSA from Alaska that we studied showed a high percentage of clindamycin resistance (57.5%). In contrast, strains of MRSA that cause community-onset skin infections elsewhere in the United States are commonly susceptible to clindamycin (*2**–**4**,**8*), although exceptions have been documented (*34*), most recently in San Francisco among men who have sex with men infected by USA300 strains (*35*). Isolates from Alaska also had a relatively low percentage of erythromycin resistance, which reflects the predominance of the ST1 background. Erythromycin-resistant MRSA strains likely have the *erm* gene, which confers inducible or constitutive resistance to clindamycin, although there are other molecular mechanisms for clindamycin resistance. Surprisingly, among the prospective isolate collection, every isolate resistant to erythromycin was also resistant to clindamycin by single-agent testing, an observation suggesting that the presumably *erm*-mediated phenotype became constitutive more often. Compared with antimicrobial drug susceptibilities among MRSA identified in Alaska in 2000, clindamycin resistance remained high but decreased slightly from 61% to 57.5%, ciprofloxacin resistance increased from 0% to 7%, and susceptibilities to other antimicrobial drugs remained similar in the prospective collection. In contrast to the situation elsewhere in much of the United States, in southwestern Alaska, clindamycin should be avoided as a first-line agent for treatment of community-onset SSTIs.

Our study was limited because the isolates we examined were from patients in 1 region and the number available in the retrospective collection was not large. The prospective collection was obtained from 1 clinic system and its community hospital, which may not be representative of other regions of Alaska. Furthermore, few clinical data were available regarding patients from whom these isolates were obtained. Our data suggest that further research is needed to clarify the enigma of nearly simultaneous emergence and high prevalence of MRSA strains with PVL toxin genes and SCC*mec* type IV elements in different predominant genotype backgrounds in different regions of the world.
